# Proteomic research in sarcomas – current status and future opportunities

**DOI:** 10.1016/j.semcancer.2019.11.003

**Published:** 2020-04

**Authors:** Jessica Burns, Christopher P Wilding, Robin L Jones, Paul H Huang

**Affiliations:** a Division of Molecular Pathology, The Institute of Cancer Research, London, SW3 6JB, UK; b Division of Clinical Studies, The Institute of Cancer Research, London SW3 6JB, UK; c Sarcoma Unit, The Royal Marsden NHS Foundation Trust, London, SW3 6JJ, UK

**Keywords:** Sarcoma, Proteomics, Targeted Therapy, Biomarkers, Drug Discovery

## Abstract

Sarcomas are a rare group of mesenchymal cancers comprising over 70 different histological subtypes. For the majority of these diseases, the molecular understanding of the basis of their initiation and progression remains unclear. As such, limited clinical progress in prognosis or therapeutic regimens have been made over the past few decades. Proteomics techniques are being increasingly utilised in the field of sarcoma research. Proteomic research efforts have thus far focused on histological subtype characterisation for the improvement of biological understanding, as well as for the identification of candidate diagnostic, predictive, and prognostic biomarkers for use in clinic. However, the field itself is in its infancy, and none of these proteomic research findings have been translated into the clinic. In this review, we provide a brief overview of the proteomic strategies that have been employed in sarcoma research. We evaluate key proteomic studies concerning several rare and ultra-rare sarcoma subtypes including, gastrointestinal stromal tumours, osteosarcoma, liposarcoma, leiomyosarcoma, malignant rhabdoid tumours, Ewing sarcoma, myxofibrosarcoma, and alveolar soft part sarcoma. Consequently, we illustrate how routine implementation of proteomics within sarcoma research, integration of proteomics with other molecular profiling data, and incorporation of proteomics into clinical trial studies has the potential to propel the biological and clinical understanding of this group of complex rare cancers moving forward.

## 1 Background

Soft tissue (STSs) and primary bone sarcomas are a group of cancers originating from the malignant transformation of multipotent mesenchymal stem cells. Sarcomas are rare accounting for less than 1% of all adult cancer diagnoses made annually with primary malignant bone tumours making up approximately 10% of all sarcoma diagnoses (1,2). Despite this common cell of origin, sarcomas represent a heterogeneous group of cancers, with over 70 different histological subtypes characterised by diverse pathologies and genetic aberrations. This biological heterogeneity is reflected clinically by notable variation in the natural history of different sarcoma subtypes and variable patterns of response to therapy (3). Accordingly, soft tissue and bone sarcoma diagnoses can range from indolent and curable neoplasms to highly lethal tumours with aggressive, metastatic and recurrent clinical phenotypes. The mainstay of treatment of curative intent for localised sarcomas is *en-bloc* surgical excision. However, in cases whereby anatomical considerations make surgical excision unfeasible, and in the presence of metastases, systemic chemotherapy or radiotherapy becomes necessary. Although the primary bone malignancies Ewing sarcoma (ES) and osteosarcoma are regarded as chemosensitive, many STS subtypes display intrinsic resistance to these current systemic regimens ([4], [5], [6]). Moreover, identification of efficacious novel therapeutics is hampered by the rarity of sarcomas, as well a “one size fits all” approach, which historically has led to the recruitment of heterogeneous patient cohorts with multiple sarcoma subtypes into clinical trials (7). Despite recent promising clinical trials in targeted therapies in select STS subtypes, such as cediranib in alveolar soft part sarcoma (ASPS), there remains an incomplete understanding of the underlying molecular drivers for the majority of sarcoma subtypes, and how these influence treatment responses (8). As such, sarcomas are diseases of unmet need both in terms of inadequate biological understanding and lack of effective therapies across all subtypes.

**Fig. 1:**
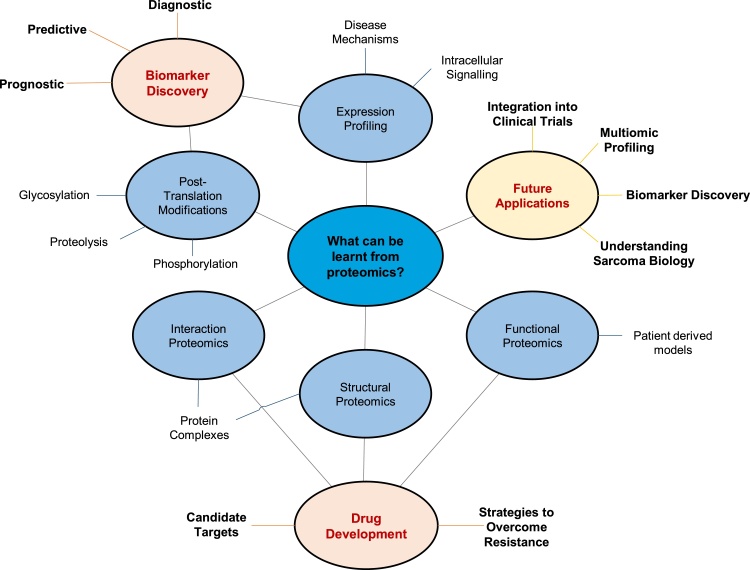
The potential benefit of using proteomic approaches in sarcoma research. Different branches of proteomics (blue) offer insights into various aspects of sarcoma development, driving clinical advances (orange) as well as offering future avenues (yellow) which are yet to be explored.

Since the completion of the human genome project, our understanding of the genetic basis driving cancer development has greatly improved. In comparison, the human cancer proteome has remained largely unexplored, and proteomic studies are few in number relative to the abundance of genomic studies (9). This genomic-proteomic discrepancy is acutely apparent in sarcomas, where proteomic studies have focused mainly on the most common subtypes, and few studies can be considered truly comprehensive. The lack of sarcoma proteomic studies can largely be attributed to disease rarity; where an inherent lack of sample availability as well as low interest by pharmaceutical companies for drug development has restricted progress. Despite these limitations, the potential for proteomic exploration of rare diseases should not be underestimated. Proteomic techniques employed for the assessment of other cancers types have provided complementary and contrasting data to their genomic counterparts. This has helped drive developments in the molecular understanding of disease, for example identifying proteomic subtypes associated with cancer behaviour, such as proliferation and invasion, and generating more robust biomarkers when utilising genomic, epigenomic and proteomic features, relative to biomarkers comprising a single data type ([10], [11], [12], [13]). Further to dissecting the molecular pathology of sarcomas, proteomics also holds promise with respect to biomarker discovery and new therapeutic target identification; both of which are notably absent in the sarcoma field. Proteomic studies aimed at identifying candidate biomarkers often reveal valuable protein identifiers for diagnosis, patient stratification, prognosis and prediction of clinical course. As a result, these studies allow for improved disease monitoring, and in turn, permit informed clinical decisions to be made; driving more favourable clinical outcomes. Moreover, proteins are the targets for a vast majority of therapeutics, and thus identification of key proteins or protein modifications mediating sarcoma progression, metastasis, and resistance to current treatments, can reveal new avenues for therapy. Aside from direct clinical benefit, given the functional role of proteins in regulating physiological and pathological processes, proteomics provides critical biological insight to improve our understanding of disease aetiology and progression (14). Such improvements in the basic biological understanding of disease will ultimately provide a solid foundation for accelerating new advances in these rare cancers (Fig. 1).

Herein, we present a comprehensive review of proteomic approaches employed within sarcoma research, and establish the current status of proteomics in both rare and ultra rare sarcoma subtypes. Further to this, we provide an outlook for the future of sarcoma proteomics, highlighting current limitations within the field, and present solutions for how these challenges may be overcome to facilitate rapid adoption by the research community.

## 2 Overview of proteomic strategies

Proteomics describes the study of entire protein complement in a system of interest; be it individual cellular components, or an entire organism. Proteomics is not limited to the study of protein abundance, but also involves analysis of protein regulation and activity. This includes, but is not limited to, the detection of protein isoforms, post-translational modifications, and protein-protein interactions. Integrated analysis of protein status in these multiple contexts provides unparalleled insight into the dynamic proteome. A number of different approaches, each with specific advantages and disadvantages, have been developed for use in cancer research. These can broadly be classified into non-mass spectrometry (MS)- and MS-based strategies (Table 1).

**Table 1:** Summary of the sarcoma proteomic studies as described herein.

	Study	Approach	Sample	Sample information	Number of proteins assessed	Key findings
**Osteosarcoma**	Kikuta K et al. [30]	2D-DIGE	Frozen tissue	6 tumours responsive to ifosamide/ doxorubicin/cisplatin & 6 not	38 differentially expressed proteins	• PRDX2 expression is increased in tumours of patients responding poorly to ifosamide, doxorubicin, cisplatin, and methotrexate
		Western blot	Frozen tissue	4 tumours	1 protein (PRDX2) assessed for validation	
	Kubota D et al. [31]	2D-DIGE	Frozen tissue	7 tumours responsive to methotrexate/ doxorubicin/cisplatin & 6 not	27 differentially expressed proteins (including PRDX2)	
	Chaiyawat P et al. [32]	2D-DIGE	Frozen tissue	4 tumours & normal soft tissue callus	29 upregulated proteins in OS compared to normal soft tissue callus	• Proteins involved in the unfolded protein response are enriched in OS tumours • ERp60 is upregulated in stage II disease, and GRP78 in patients poorly responsive to treatment
		Western blot	Cell line	6 primary cell lines	3 proteins (GRP78, GRP94, ERp60) for validation	
		Western blot	Frozen tissue	9 tumours	3 proteins (GRP78, GRP94, ERp60) for validation	
	Gemoll T et al. [3]	2D-DIGE	Cell line	CRL11372 (osteoblasts), CRL7023/ 7134/7140 (OS) & CRL7585/7631/ 7645 (OS metastases)	13 upregulated and 4 downregulated proteins in all OS and OS metastasis lines	• CTSD expression is increased in OS relative to normal bone, and in pulmonary metastases relative to primary-site OS
		IHC	FFPE tissue	TMA of 4 normal bone tissue, 17 OS & 5 OS pulmonary metastasis tumours	1 protein (CTSD) assessed for validation	
**Ewing sarcoma**	Gurpide JM et al. [40]	2D-DIGE	Cell line	TC-71 & TC-71 (*EWS-FLI1* knockdown)	39 differentially expressed proteins	• TRAF6 is a hub protein in cells not expressing *EWS/FLI1, and is* localised around inflammatory cells.
	Hawkins AG et al. [41]	LC-MS/MS	Cell line	TC32 & CHLA10 secretome (with and without Wnt3a supplemented)	2336 (TC32) & 847 (CHLA10) proteins identified, of which 543 & 259 were annotated as secreted	• The basal ES secretome is enriched for IGF transport proteins, and the wnt-dependent secretome enriched in ECM-regulators.
**Gastrointestinal stromal tumour**	Ichikawa H et al. [55]	ESI-MS/MS	Frozen tissue	4 stomach & 4 intestinal tumours	2555 proteins identified (54 differentially expressed between stomach and intestinal lesions)	• PML expression is significantly lower in intestinal GIST • There is a higher 5-year recurrence-free survival in PML positive cases (91.7%) compared to PML negative cases (60.1%)
		IHC	FFPE tissue	128 stomach, 15 intestinal & 13 tumours of 'other' sites	1 protein (PML) assessed for validation	
	Suehara Y et al. [56] **[A]** Kikuta K et al. [61] **[B]**	2D-DIGE	Frozen tissue	8 tumours with metastases 1 year post-surgery & 9 with none	38 proteins identified (25 differentially expressed)	• Pfetin and DDX39 expression is significantly higher in tumours from patients with metastases 1 year post-surgery • High pfetin expression correlates with increased tumour size, mitotic index and degree of differentiation [A] and high DDX39 expression correlates with shorter surivival **[B]**
		IHC	FFPE tissue	210 **[A]** & 72 **[B]** tumours	2 proteins (pfetin **[A]**, DDX39 **[B]**) assessed for validation	
	Liu Y et al. [62]	LC-MS/MS	Frozen tissue	3 low, 5 intermediate & 5 high risk tumours (& matched normal tissue)	9177 proteins identified (131 were differentially expressed between risk groups)	• High PTPN1 expression is associated with low risk GIST cases, and correlates with increased disease-free survival
		IHC	FFPE tissue	131 tumours of mixed risk status	1 protein (PTPN1) assessed for validation	
	Da Riva L et al. [69]	MALDI-MS	Frozen tissue	1 untreated, 7 IM responsive & 8 IM non-responsive tumours	39 proteins identified	• There is a significantly increased expression of stromal SCGF in IM-responsive GIST tumours
		IHC	FFPE tissue		1 protein (SCGF) assessed for validation	
	Takahashi T et al. [71]	LC-MS/MS	Cell line	GIST-T1 (IM sensitive) & GIST-T1-R (IM resistant)	171 phosphosites across 134 proteins (11 with increased pY after IM treatment)	• FAK and FYN suppression in GIST cells increases IM sensitivity • FAK and FYN suppression in IM resistant GIST cells triggers apoptotic cell death
	Nagata K et al. [72]	LC-MS/MS	Cell line	GIST882 (IM sensitive) & GIST882-R (IM resistant)	3468 phosphopeptides identified	• Phosphorylation of KIT and EGFR is upregulated in IM resistant cells • Treatment with EGFR inhibitor, gefitinib, sensitises resistant cells to IM
	Berglund E et al. [73]	LC-MS/MS	Cell line	GIST882 (IM sensitive) secretome	531 identified	• The secretome of IM treated cells is enriched for protein translation, transcription, and exosomal components
	Atay S et al. [74]	LC-MS/MS	Cell line	GIST882 (IM sensitive) & GIST-T1 (IM sensitive) exosomes	1060 proteins identified as common to all assessed exosomes	• GIST exosomes (both cell line and patient derived) are rich in KIT protein and IM response proteins
		Western blot	Plasma	Patient derived exosomes	32 proteins assessed for validation	
**Liposarcoma**	Cervi D et al. [87]	SELDI MS	Platelets & plasma	Xenograft mice (parent cell line SW872)	1 protein (PF4) assessed in depth	• Pro-angiogenic PF4 is detected at high levels in platelets of LPS mice from day 19 post-implantation (non-palpable tumour stage), and not in non-tumour bearing mice
	McClain CM et al. [88]	2D-DIGE	Frozen tissue	8 Lipoma, 8 ALT & 3 DDLPS tumours	6 differentially expressed proteins in WDLPS areas of DDLPS, compared to ALT	• Expression of SELENBP1 is significantly lower in WDLPS compared to ALT
		IHC	FFPE tissue	30 ALT & 28 DDLPS tumours	1 protein (SELENBP1) assessed for validation	
**Leiomyosarcoma**	Kirik U et al. [96]	2D-DIGE	Frozen tissue	38 LMS & 16 UPS tumours	778 proteins identified	• 4 proteomic subgroups were identified (1 UPS-enriched, 3 comprising LMS cases)
		LC-MS/MS	Frozen tissue	5 tumours from each subgroup identified by 2D-DIGE		• The expression levels of 3 proteins (VINC, COL6A3, MYH11) were identified as capable of discriminating between the 4 subgroups identified
	Lin ST et al. [98]	2D-DIGE	Cell line	MES-SA cells (uterine LMS)	1,755 protein spots identified	• The expression of 87 proteins alters upon short-term, acute doxorubicin treatment.
	May EWS et al. [99]	2D-DIGE	Cell line	MES-SA cells & MES-SA/DxR cells (doxorubicin resistant)	208 differentially expressed proteins	• MES-SA/DxR cells display an upregulation of metabolic pathway proteins and a downregulation of proteins involved in cell proliferation, gene regulation, and signal transduction.
	Yang J et al. [108]	RPPA	Frozen tissue	31 LMS, 38 GIST tumours & 24 matched normal tissue (TMA constructed for IHC)	7 MET/EMT related proteins assessed	• Higher expression of E-cadherin and lower expression of Slug correlates with improved overall survival in LMS.
		IHC	FFPE tissue		2 proteins (E-cadherin & Slug) assessed	
**Malignant rhabdoid tumours**	Darr J et al. [115]	LC-MS/MS	Cell line	Cell lines 167, 365, & 365 expressing *Smarcb1*	3655 proteins identified (407 with differential phosphorylation level)	• Smarcb1-deficient cells show enrichment and increased phosphorylation of EGFR and its downstream effectors. • PhosphoAkt is reduced by EGFR inhibition.
	Wong J et al. [116]	LC-MS/MS	Cell line	A204, A204/PazR (pazopanib resistant) & A204/DasR (dasatinib resistant)	Focused on the phosphorylation of 2 proteins: PDGFRα and FGFR1	• Cells resistant to PDGFRα inhibition are sensitive to FGFR inhibition; dual inhibition of both receptors can overcome resistance.
	Vyse S et al. [117]	LC-MS/MS	Cell line	A204, A204/PazR (pazopanib resistant) & A204/DasR (dasatinib resistant)	7548 phosphosites quantified	• DasR cells are enriched for phosphorylation of IGF-1R pathway.
**Myxofibrosarcoma**	Kikuta K et al. [124]	2D-DIGE	Frozen tissue	6 invasive & 5 non-invasive tumours	47 differentially expressed proteins between invasive & non-invasive tissue	• DCBLD2 expression is upregulated in more invasive tumours, and its expression has high specificity (87.5%) as a predictor for invasive phenotype.
		IHC	FFPE tissue	21 tumours of mixed invasiveness	1 protein (DCBLD2) assessed for validation	
**Alveolar soft part sarcoma**	Kubota D et al. [131]	2D-DIGE	Frozen tissue	12 tumours (& matched normal tissue)	145 differentially expressed proteins between ASPS tumour & matched normal tissue	• SET is overexpressed in tumour tissue compared to normal, and its expression is central to proliferation, migration and invasion.
		IHC	FFPE tissue	15 tumours (including those assessed by 2D-DIGE)	1 protein (SET) assessed for validation	
**Integrative studies**	Abeshouse A et al. [93]	RPPA	FFPE tissue	60 LMS, 46 DDLPS, 41 UPS, 15 MFS, 6 SS & 5 MPNST tumours	192 proteins assessed	• LMS has a distinct proteomic signature compared to other subtypes, with lower apoptotic activity, higher PI3K/AKT activity, and higher hormone receptor expression.
	Lou S et al. [132]	MALDI-MS	Frozen tissue	53 OS, LMS & UPS tumours	9 proteins (associated with poorer survival)	• OS, LMS and UPS have distinct proteome profiles. • 9 proteins are associated with poorer survival in both LMS and UPS.
	Lou S et al. [133]	MALDI-MS	Frozen tissue	10 MFS, 7 OS, 8 LMS & 8 UPS tumours	3 metabolites (associated with poorer survival)	• OS, LMS, UPS, and MFS have distinct metabolite profiles. • Inositol cyclic phosphate and carnitine correlate with poorer OS and MFS.

### 2.1 Non-mass spectrometry-based proteomic approaches

The non-MS proteomic approaches employed in sarcoma research are near exclusively microarray-based methods, most commonly, reverse-phase protein microarrays (RPPAs). RPPAs require immobilisation of tumour lysates onto a microarray surface, followed by probing with an antibody targeting a protein under investigation to quantitatively assess said protein levels across hundreds of samples simultaneously (15). The converse strategy, known as antibody arrays can also be performed. This approach involves the immobilisation of a panel of antibodies against a range of protein targets and subsequent probing with lysate from a tissue sample of interest to simultaneously evaluate the levels of multiple proteins in one specimen. Microarrays are inherently high-throughput, and allow for rapid and cost-effective proteomic profiling. Critically, they require minimal amounts of input material, and are thus attractive for use in ultra-rare sarcoma subtypes for which limited sample material is available. Although non-MS based methods have provided, and continue to provide, valuable biological and clinical insights into sarcomas, their dependence on antibodies has its limitations. For one, antibodies are not readily available for all proteins, nor are all those available truly specific to the target protein (16). As a result, achieving a proteome depth beyond several hundred proteins using microarray strategies is unachievable. Further to this, *a priori* knowledge of the protein(s) of interest is required, and thus such approaches cannot be utilised for a truly unbiased, discovery-based proteomic assessment.

### 2.2 Mass spectrometry-based proteomic approaches

In this section, we provide a brief description of the MS strategies commonly used in sarcoma proteomic studies that have been reported thus far. A detailed discussion of the fundamentals of MS is beyond the scope of this review, but readers are directed to excellent reviews on this topic (17,18). In contrast to non-MS-based approaches, MS-based methods are unbiased and provide more sensitive and accurate identification of proteins. As a result, MS-derived proteomic profiles are far more comprehensive in depth of proteome coverage. However, major limitations persist in the use of MS methods. Most notably, these approaches are also notoriously low throughput and despite advancements in multiplexed isobaric labelling techniques, MS analysis is unlikely to achieve the sample throughput currently available with microarray strategies.

Clinical proteomics often involves the study of complex biological samples, and therefore to improve proteome coverage prior to MS analysis, fractionation of peptides post protein digestion is frequently performed. Common methods for such peptide fractionation include reverse-phase liquid chromatography, isoelectric focusing, strong cation exchange, and high pH fractionation, the advantages and disadvantages of which have been discussed at length elsewhere (19,20). Fractionation can also be performed prior to protein digestion, often by polyacrylamide gel electrophoresis (PAGE), and is commonly used for STS proteome assessment by two-dimensional difference electrophoresis (2D-DIGE). Samples for 2D-DIGE analysis are labelled with excitable fluorescent dyes and separated using PAGE across two dimensions. Gel scanning at each dye-specific wavelength reveals differential protein spots between samples, which are subsequently digested ‘in-gel’, retrieved, and identified by MS (21).

Post sample fractionation, two main types of MS are utilised: electrospray ionisation (ESI), or matrix-assisted laser desorption/ionisation (MALDI). Irrespective of method of ionisation, protein identification is then performed by analysis and interpretation of the resultant mass spectrum generated. Further to achieving high confidence protein identification using ESI- or MALDI-MS, accurate quantitation is also central to successful proteomic experiments. Several approaches have been established to achieve this, however label-free quantitation and isobaric labelling have been most frequently employed in sarcoma proteomic studies. In isobaric labelling, the two major variants used are, isobaric tags for relative and absolute quantitation (iTRAQ) and tandem mass tagging (TMT) wherein samples are labelled with isotopically differing tags, pooled and then run in the mass spectrometer. Upon fragmentation-induced tag cleavage, reporter ions are generated in the mass spectrum and used for peptide and protein quantitation (22,23).

The variety of proteomic methods currently available allows for proteome assessment in many different sample types, be it cell line-derived material, frozen tissue or archival formalin-fixed paraffin-embedded (FFPE) tissue. Given the biological, chemical, and physical differences in these distinct sample types, it is no surprise that some proteomic approaches are better suited for the assessment of specific sample types over others. This is reflected in the current status of proteomic research where almost all MS studies in sarcoma to date have been performed on either frozen tissue or cell line-derived material. This can in part be explained by the ease of protein extraction from both frozen tissue and cell lines. In contrast, protein retrieval from FFPE tissue for MS analysis is challenging due to the presence of both paraffin and formalin-induced crosslinks which hinders effective proteomic characterisation. Reflective of these challenges, FFPE tissue is currently most routinely used for immunohistochemistry (IHC) assessment to provide spatial resolution of specific protein analytes to complement comprehensive proteomic screens rather than for MS analysis itself.

## 3 Current status of proteomic research in sarcomas

### 3.1 Osteosarocoma

Osteosarcomas are derived from primitive bone-forming mesenchymal stem cells, and are the most common primary malignant bone tumours. Osteosarcomas have an approximately equal gender distribution but a bimodal age distribution, with incidence peaks before the age of 24 and after the age of 60 years, and an incidence of around 4.2 cases per million of the population per year in these age groups (24). Histologically, conventional osteosarcoma is characterised by spindle to polyhedral cells, containing pleomorphic and hyperchromatic nuclei. Osteosarcoma cells are engaged in the production of extracellular matrix, with osteoid production directly from tumour cells a prerequisite for diagnosis. Further detail on the histological variants of osteosarcoma is beyond the scope of this review but has been covered in detail elsewhere (25). Molecular analysis has revealed that osteosarcomas are genomically unstable with complex karyotypes characterised by chromosomal instability and high levels of structural variants and copy number changes (26). Furthermore in paediatric osteosarcoma, single-nucleotide variants exhibited kataegis, a pattern of localized hypermutation, with recurrent mutations most frequently observed in the tumour protein p53 (*TP53)* gene, and also in retinoblastoma protein-1 (*RB1)*, alpha thalassemia/mental retardation syndrome X-linked chromatin remodeler (*ATRX)*, and discs large MAGUK scaffold protein-2 (*DLG2)* (27). Survival following osteosarcoma improved considerably from a five-year OS rate of 19.3% in the 1950s to around 60% in the 1980s, attributable to advances in multi-agent systemic chemotherapy (28,29). However, over the past three decades although the rate of limb salvage versus amputation has improved, likely due to improved surgical techniques, implant designs and disease awareness, the rate of OS at 5 years has plateaued.

Several therapeutic avenues are available to osteosarcoma patients, including chemotherapy. However, patient response to treatment varies greatly and there currently exists no method for predicting patient response. Two studies have addressed the lack of biomarkers for therapy response by assessing chemonaïve tumour tissue using proteomic strategies. Kikuta et al. used 2D-DIGE to identify biomarkers of response to chemotherapy with ifosamide (IFO), doxorubicin, and cisplatin (CDDP) (30). 6 tumour biopsy samples from patients with a poor pathological response to chemotherapy (defined as <90% necrotic tissue) and 6 tumour biopsy samples from patients with a good response (GR; defined as >90% necrotic tissue) were profiled. Thirty eight differentially expressed proteins were identified, including peroxiredoxin 2 (PRDX2), a protein that was found to be highly expressed in those with a poor response. The observed association of PRDX2 in those with a poor response was subsequently confirmed by western blot in an independent cohort of 4 further osteosarcoma cases. In another study, Kubota et al. compared poor and favourable responders to identify predictors of response to chemotherapy with methotrexate (MTX), doxorubicin, and CDDP (31). Using 2D-DIGE to interrogate the proteomes of pre-treatment tissue from 6 poor responders and 7 GRs, the authors revealed 27 proteins whose expression significantly differed between responder groups. Consistent with the previous study by Kikuta et al., PRDX2 was similarly found to be upregulated in those with a poor response. To further investigate the impact of PRDX2 expression on cell response to MTX, doxorubicin, and CDDP, cell viability assays were performed using the MNNG/HOS, MG63, and 143B osteosarcoma cell lines. These experiments found that cell viability in all cell lines to be significantly lower when PRDX2 was silenced by RNAi. Moreover, in PRDX2 knockdown cells, functional assays revealed a reduction in cell proliferation, invasion, and migration, as characterised by growth, invasion and wound healing assays respectively. Collectively, these two studies present PRDX2 expression as a candidate predictive biomarker for patient stratification to chemotherapy and may represent a causative driver of more aggressive disease in osteosarcoma.

In addition to the proteomic investigation of osteosarcoma for biomarker discovery, global proteome profiling has been employed to better understand osteosarcoma oncogenesis and reveal novel therapeutic targets. Chaiyawat et al. profiled the proteomes of tumour tissue from 4 chemonaïve patients by 2D-DIGE (32). Comparison with normal soft tissue callus highlighted 29 proteins as significantly upregulated in osteosarcoma, which were ontologically enriched for metabolic processes, biological regulation, and protein binding activity. Subsequent integration of ontology and network analysis revealed enrichment of the unfolded protein response (UPR) components, including 78 kDa glucose-regulated protein (GRP78) endoplasmin (GRP94), calreticulin (ERp60) and prelamin-A/C. Validation of these candidate proteins was undertaken by western blot in 6 primary osteosarcoma cell lines. In line with proteomics data, upregulation of GRP78, GRP94 and prelamin-A/C in osteosarcoma cells, compared to primary osteoblastic cells, was observed. Further to this, tissue from an independent cohort of 9 osteosarcoma patients was assessed for UPR protein expression by western blot, and integrated with disease stage. Of the 9 patients, three had stage IIB disease and had a good response to chemotherapy (tumour necrosis >90%), three were stage IIB and had a poor response (tumour necrosis <90%), and three had stage III (metastatic) disease. Comparison with normal soft callus and integration of clinical staging revealed GRP94 upregulation in osteosarcoma irrespective of status. ERp60 upregulation was observed in stage II disease compared to normal soft callus, and GRP78 was found to be upregulated in poor responders, compared to GRs. Taken together, these findings suggest therapeutic targeting of the UPR pathway may be a viable approach for osteosarcoma management and targeting components of the UPR may prove beneficial to patients refractory to conventional chemotherapy.

Despite often successful management of primary osteosarcoma, many patients subsequently develop metastatic lesions, most commonly in the lung. The presence of such pulmonary metastases vastly worsens prognosis, and is the most common cause of death in osteosarcoma. The precise molecular mechanism for the occurrence of osteosarcoma metastasis however remains unclear. To identify potential drivers mediating metastasis, proteomic profiling by 2D-DIGE has been performed to compare the foetal osteoblast cell line CRL11372, osteosarcoma cell lines CRL7023, CRL7134 and CRL7140, and osteosarcoma pulmonary metastasis cell lines CRL7585, CRL7631, and CRL7645 (33). This yielded 13 proteins consistently upregulated and 4 consistently downregulated in osteosarcoma and metastatic osteosarcoma cells compared to osteoblasts. Ingenuity pathway analysis revealed a single network comprising 13 of the 17 proteins identified, and highlighted V-myc myelocytomatosis viral oncogene homolog (MYC), stathmin1 (STMN1), cathepsin D (CTSD), and TP53 as hub nodes. Of these, STMN1 and CTSD were selected for further validation. Western blotting of CTSD correlated with 2D-DIGE data, illustrating increased expression in osteosarcoma and osteosarcoma metastases. However, the reduction in STMN1 expression in osteosarcoma cells observed by 2D-DIGE could not be reproduced. CTSD expression was further validated by IHC in a tumour microarray (TMA) comprising 4 normal bone tissue samples, 17 osteosarcoma samples, and 5 osteosarcoma pulmonary metastasis samples. Through IHC scoring, CTSD expression was confirmed as increased in osteosarcoma relative to normal bone, and furthermore was significantly higher in pulmonary metastatic lesions than in primary-site osteosarcoma. As such, CTSD expression was revealed as a consistent differentiator between all three groups, and has been hypothesised as a driver of osteosarcoma oncogenesis and metastasis. In addition to being a marker for metastatic lesions, therapeutic targeting of CTSD may be a promising, novel avenue for osteosarcoma treatment which may yield favourable responses in metastatic disease.

### 3.2 Ewing sarcoma

ES is the second most common primary malignancy of the bone, and occurs in children and young adults with a peak incidence at 15 years of age. ES has an aggressive phenotype, with a propensity for local recurrence and early haematogenous metastases, most commonly to the lung. Histologically, ES are composed of sheets of small round cells, with a high nuclear to cytoplasmic ratio, little mitotic activity, and usually contain periodic-acid-Schiff-positive granules (34). ES is a translocation driven sarcoma; t(11,22)(q24;q12) results in the formation of the *EWSR1-*FLI1 proto-oncogene (*FLI1*) fusion gene in 85% of cases; t(21,22)(q22;q12) in the *EWSR1-*ETS transcription factor ERG (*ERG)* fusion gene in 10%; and the remaining 5% of cases are characterised by other translocations all resulting in a fusion gene containing a portion of the *EWSR1* gene (35,36). The fusion proteins resulting from these translocations act as aberrant transcription factors due to retention of the N-terminal potent transcriptional regulatory domain of *EWSR1.* ES oncogenesis is then driven by upregulation of downstream target genes including nuclear receptor subfamily 0 group B member 1 (*NR0B1*), GLI family zinc finger 1 (*GLI1)* and the forkhead box (*FOX)* group of transcription factors (37). ES is both radio- and chemo-sensitive, and its clinical management is reliant on multimodal treatment. The current treatment paradigm involves induction chemotherapy, surgical resection and post-operative chemotherapy with or without radiation. The degree of tumour necrosis observed in the resection specimen, in response to induction chemotherapy, has been associated with improved OS although this remains an issue for debate, whilst post-operative radiotherapy has demonstrated a significant reduction in local recurrence compared to surgery alone (38,39).

The *EWS-FLI1* fusion is a key driver in ES oncogenesis. As such downstream effectors and target proteins of *EWS-FLI1* are likely implicated in disease pathogenesis and are thus of interest to the research field. To identify such candidate proteins, comparative proteomic profiling of the ES cell line TC-71 and an *EWS-FLI1* knockdown TC-71 cell line variant known as shEWS-FLI1 has been performed using 2D-DIGE (40). This study identified 25 proteins as differentially upregulated in shEWS-FLI1 cells, and 14 proteins as downregulated relative to *EWS-FLI1* expressing cells. Gene ontology of the differentially expressed proteins showed an enrichment of DNA and RNA processing proteins in the shEWS-FLI1 cells, which is consistent with the transcriptional role of *EWS-FLI1*. Interestingly, network analysis revealed TNF Receptor Associated Factor 6 (TRAF6), an ubiquitin ligase which activates NF-kappa-B and JUN, as a hub node upregulated upon *EWS-FLI1* knockdown. TRAF6 expression levels were assessed further by IHC in an independent cohort of 42 ES samples, which highlighted an absence of TRAF6 within the ES tumour cells and provided spatial resolution of TRAF6 localisation in tumours. This analysis indicated TRAF6 as predominantly localised around inflammatory cells which implicates a potential role of inflammation in contributing to the oncogenesis of ES.

In addition to the study of the tumour cellular proteome, others have also shown that ES cells also have a distinct secretome (41). In a study by Hawkins, et al., the conditioned media of 2 ES cell lines, TC32 and CHLA10, were isolated under serum-starved conditions and profiled using MS. In total, this study yielded 2336 proteins from TC32 cells and 857 from CHLA10 cells. Identified proteins were mapped to the Human Protein Atlas and those annotated as secreted, 543 from TC32 cells and 259 from CHLA10 cells, were classified as tumour secretome components. Integration of these datasets finds that over 33% of the TSC32 secretome was also detected in the CHLA10 secretome, alluding to the presence of a common ES secretome identified irrespective of cell line differences. Considering aberrations in insulin-like growth factor (IGF) signalling has been reported in ES, it was notable that the common secretome included an enrichment of proteins involved in insulin-like growth factor (IGF) transport (42).

Previous reports have revealed a cancer stem cell (CSC) population within ES tumours, identifiable by expression of the cell surface receptor leucine-rich repeat-containing G-protein coupled receptor 5 (LGR5). LGR5 is a target of Wnt3a and R-spondin-1, which once bound interacts with other Wnt receptors and triggers their internalisation and degradation, thus potentiating Wnt/beta-catenin signalling. The addition of the Wnt3a ligand to ES cells is able to simulate the high Wnt/beta-catenin signalling normally observed in CSCs (43). Hawkins et al., sought to assess the effects of supplementing Wnt3a on the ES secretome. This study resulted in the identification of 33 and 16 differentially expressed proteins when Wnt3a was added to TCS32 and CHLA10 respectively. Gene set enrichment analysis showed that the Wnt-dependent secretome was enriched for proteins regulating extracellular matrix (ECM) organisation and ECM-cell interactions. These include several proteins with a known role in collagen and ECM organisation such as Transforming Growth Factor Beta Induced (TGFBI), Matrilin 3 (MATN3), A Disintegrin and Metalloproteinase domain-containing protein 9 (ADAM9), Matrix Metalloproteinase 19 (MMP19), and Tenascin C (TNC). Taken together, these data indicate that ES tumour cells can modulate the tumour microenvironment (TME) by altering the ECM composition in response to Wnt ligand.

### 3.3 Gastrointestinal Stromal Tumours

Gastrointestinal stromal tumours (GISTs) are the most common mesenchymal tumours of the gastrointestinal tract, occurring anywhere from oesophagus to rectum, with the stomach being the most common location (60% of GIST) followed by the small intestine (25%) (44). They tend to occur in the elderly with a peak incidence in the seventh decade of life, and are slightly more common in males (45). Histologically, GISTs demonstrate a broad spectrum of morphology, ranging from a bland spindle cell proliferation to highly cellular epithelioid tumours with nuclear pleomorphism (46). Oncogenic mutations in GIST have been well described, and it is now established that 75% of GISTs harbour a gain of function mutation in the KIT proto-oncogene (*KIT)* gene. KIT is a member of the type III receptor tyrosine kinase family, and the mutated gene leads to ligand-independent activation and subsequent stimulation of downstream intra-cellular signalling pathways (47). It has also been demonstrated that *KIT* mutational status has prognostic significance, with exon 9 mutations having a higher risk of progression relative to exon 11 mutations, whilst exon 11 deletions have a higher rate of relapse relative to other types of exon 11 mutations (48). Aside from *KIT* mutations, a further 10% of GISTs harbour mutations in the platelet derived growth factor receptor alpha (*PDGFRA*) gene. PDGFRA is also a member of the type III receptor tyrosine kinase family, and again mutation leads to constitutive activation of the receptor triggering downstream intracellular signalling pathways. Finally, 15% of patients with GISTs do not have a detectable mutation in either *KIT* or *PDGFRA*. These tumours are a genomically heterogeneous group with a variety of genetic anomalies previously reported, including mutations in B-Raf proto-oncogene (*BRAF)*, succinate dehydrogenase (*SDH)* and neurofibromin 1 (*NF1),* however they do demonstrate phosphorylated KIT although the mechanisms behind this are unclear ([49], [50], [51]). The understanding of the mutations driving GIST led to trials of the tyrosine kinase inhibitor imatinib mesylate (IM) for patients with GIST. Initially developed as a treatment for chronic myeloid leukaemia due to its activity against the fusion protein breakpoint cluster region-Abelson murine leukaemia (BCR-ABL), the structural similarity between ABL and KIT led to pre-clinical and subsequent clinical trials confirming IM activity in GIST (52). IM has consequently transformed the management of advanced GIST, with a rate of overall survival (OS) at 5 years of over 60% for patients treated with IM versus 35% for conventional chemotherapy (53,54). In this section of the review, we will assess the role proteomics has played in stratifying patients most likely to respond to IM and other tyrosine kinase inhibitors, as well as exploring mechanisms for primary and acquired drug resistance.

The anatomical location of GIST lesions is frequently indicative of clinical course. Protein signatures mapped to anatomically distinct GISTs could provide a basis for dissecting location-specific molecular profiles, thus improving the understanding of disease initiation and progression. Comparative proteomic profiling by ESI-MS/MS was performed on 4 surgically resected stomach GISTs, of which 2 were *KIT*/*PDGFRA* wild type and 2 harboured *KIT* mutations, and 4 surgically resected small intestine GISTs, all possessing *KIT* mutations (55). This analysis revealed 54 proteins that significantly differ in expression between these 2 anatomical locations. Of these proteins, 29 were reported as upregulated in the intestinal lesions, and 25 as upregulated in the stomach. In parallel, transcriptomic analysis of an expanded cohort was performed and the resultant data integrated with proteomics data to reveal 18 proteins that were concordant with gene expression levels. Of note, the tumour suppressor protein, promyelocytic leukemia (PML), was identified as expressed at significantly lower levels in intestinal GIST. Low PML expression in intestinal tumours was further validated by immunohistochemistry (IHC) on an independent cohort of 156 GIST cases (15 intestinal, 128 stomach, and 13 other), which identified all intestinal GIST cases as PML negative. Subsequent integration of validation cohort IHC data with clinical data found a higher 5-year recurrence-free survival rate in cases detected as PML positive (91.7%; stomach and other GIST cases) than those detected as PML negative (60.1%; intestinal and other GIST cases).

Identification of biomarkers predictive of high relapse risk in GIST will improve disease monitoring and clinical management. To discover new predictive biomarkers of GIST relapse, Suehara et al. used 2D-DIGE to profile surgically resected GIST tumours from 8 patients who developed metastatic lesions within 1 year post-surgery (P-GIST) and 9 patients with no evidence of metastasis 2 years post-surgery (G-GIST) (56). A single P-GIST case and 3 G-GIST cases were *KIT* wildtype, whilst the remainder of the cohort possessed a *KIT* mutation. This analysis revealed 25 proteins that differed significantly in expression between the P-GIST and G-GIST groups. Of note, the expression of the potassium channel tetramerization domain containing 12 (pfetin) protein, observed to be significantly higher in the G-GIST group, was assessed further by the investigators. IHC analysis of pfetin in an expanded and independent cohort of 210 GIST samples was performed to validate the initial proteomic analysis. Positive pfetin expression in this cohort was shown to correlate with several pathological features indicative of a poorer prognosis such as increased tumour size, increased mitotic index, and an increased degree of differentiation. Pfetin expression was statistically associated with risk classification, however this significance was attributable to low-risk (G-GIST) cases only. A pfetin negative classification was reported in 91% of low risk cases, however only 50% of high risk cases were pfetin positive. Thus, as a predictor for disease relapse, the utility of pfetin expression can only be seen in identifying patients at low risk. Several independent studies have since also reported pfetin association with a favourable prognosis in GIST ([57], [58], [59], [60]).

Further to this, the same sample cohort of 8 P-GIST and 9 G-GIST cases was reassessed by Kikuta et al. (61). As before, 25 proteins that were found to be significantly different between the P-GIST and G-GIST groups. DDX39, an ATP-dependent RNA helicase was shown to be upregulated in the P-GIST cohort and selected for further validation. IHC analysis on an independent cohort of 72 GIST samples confirmed the initial association observed between DDX39 and prognosis. Within the IHC validation cohort, samples were categorised as DDX39 strong or weak. DDX39 strong cases showed consistently higher probability of shorter survival, when compared to DDX39 weak cases, suggesting that high DDX39 expression is a predictor for high metastatic potential and, by extension, poor clinical outcome. Despite studies highlighting both DDX39 and pfetin expression as predictive of GIST recurrence, their utility in the clinic is yet to be realised. Further assessment in the prospective setting is required but these studies illustrate the potential of proteomics to identify biomarkers for risk stratification to identify high-risk patients likely to benefit from adjuvant therapy.

Further to the identification of DDX39 and pfetin as candidate drivers of disease relapse in GIST, protein phosphatases have also been hypothesised as critical modulators of tumour recurrence. Recently, a comprehensive and quantitative proteomic profiling of 13 GIST specimens with matched normal tissue was performed (62). The cohort assessed comprised samples of varied recurrence-risk subgroups; three low, 5 intermediate, and 5 high as determined by National Institute of Health consensus criteria. This study found that proteins enriched for the spliceosome pathway were upregulated in tumour samples compared to normal tissue. Conversely, proteins enriched in metabolic pathways were observed to be downregulated in tumour samples. Further to this, comparison between the different risk subgroups highlighted 131 proteins as significantly differentially expressed, of which, high expression levels of protein-tyrosine phosphatase 1B (PTPN1) showed proportionally higher association with lower risk cases. Validation by IHC on an expanded cohort of 131 GIST cases subsequently confirmed this observation. Integration with clinical data indicated that high PTPN1 was associated with increased disease-free survival.

Although the introduction of IM has helped transform patient outcomes in GIST, resistance develops in the majority of patients within 2 years of treatment, largely through the acquisition of secondary resistance mutations in *KIT* and *PDGFRA* ([63], [64], [65], [66], [67], [68]). In a number of studies, proteomics has been employed to better understand the mechanistic basis of how IM elicits its therapeutic effects and to shed further light on how resistance to IM develops in GIST. Da Riva et al. undertook a proteomic analysis in GIST tissue specimens to identify mechanisms determining response to IM (69). Comparisons were performed on 1 untreated GIST case (*KIT* mutation present), 7 treated cases responsive to IM (all possessing a *KIT* mutation) and 8 non-responsive cases (1 *KIT wild-type*, 5 harbouring a *KIT* mutation and 2 a *PDGFRA* mutation). Using MALDI-MS, the authors showed that there was a significant increased expression of stem cell growth factor (SCGF) in cases that were responsive to IM. IHC assessment of the same cases showed that high SCGF expression was restricted to the stromal compartment, supporting previous observations of SCGF secretion from dendritic cells in the stromal compartment (70). Taken together, the findings from this study suggests that the immune microenvironment may be a critical mediator of IM response in GIST.

The modulation of cellular kinome activity in response to c-KIT inhibition by IM has been interrogated by MS. Using ITRAQ labelling and immunoenrichment of phosphotyrosine peptides in the IM sensitive cell line GIST-T1, Takahashi et al. employed phosphoproteomics to evaluate changes in tyrosine phosphorylation levels upon IM treatment (71). The authors showed that 11 proteins increased tyrosine phosphorylation levels after 72 hours of IM treatment. Two kinases, focal adhesion kinase (FAK) and FYN, were selected for further assessment for their contribution to IM sensitivity. Upon genetic suppression of FAK and FYN expression by RNA interference (RNAi), GIST-T1 cells displayed increased drug sensitivity to IM. To further investigate the impact of these proteins on drug resistance, GIST-T1 IM resistant cells were derived from a resistant clone that arose during long-term culture of GIST-T1 cells in the presence of IM. Inhibition of FAK phosphorylation using the selective inhibitor TAG372 in these GIST-T1 IM resistant cells triggered apoptotic cell death. The study by Takahashi et al. highlights that pharmacological inhibition of FAK and FYN activity may be candidate salvage therapy approaches for overcoming resistance to IM in GIST. Nagata et al. performed a comprehensive serine, threonine and tyrosine phosphoproteomic profiling of the IM sensitive and KIT activated GIST882 cell line using MS (72). For comparative analysis, IM resistant GIST882 variant cells (GIST882-R) that had acquired drug resistance by long-term dose escalation treatment with IM were used. The matched pair of cell lines were subjected to phosphoproteomic profiling in the presence and absence of IM. This analysis revealed that the phosphorylation of KIT, and another tyrosine kinase, the epidermal growth factor receptor (EGFR) were significantly upregulated in IM resistant cells. Moreover, signalling molecules downstream of both kinases, such as ERK1/2 and JNK2 were also observed to have upregulated phosphorylation levels, alluding to augmented signalling through both the KIT and EGFR signalling axes. Treatment of GIST882 resistant cells with the EGFR inhibitor, gefitinib, sensitised the cells to IM, demonstrating that upregulation of EGFR signalling plays a role in the development and maintenance of IM resistance.

In addition to profiling the phosphoproteome of GIST882 cells, proteomic profiling of its secretome has also been undertaken in a separate study by Berglund et al. (73). LC-MS/MS analysis of condition media from these cells identified 375 proteins under serum-starvation conditions and 555 proteins when glucose was added to the tissue culture media. To probe the impact of KIT kinase activity on secretome composition, GIST882 cells were subjected to treatment with IM. Comparative assessment of the secretomes of IM-treated and untreated cells revealed ontological enrichment for proteins involved in the regulation of protein translation and transcription as well as protein components regulating exosome release in the IM treated cells. Moreover, interrogation of exosome databases revealed that the vast majority of secretome-identified proteins in IM treated cells were of exosomal origin. Subsequently, Atay et al. assessed the composition of GIST derived exosomes (GDEs) of GIST882 cells and GIST-T1 cells by LC-MS/MS (74). This study revealed exosomes to be rich in KIT protein expression and IM response proteins such as Sprouty homolog 4 (SPRY4) and surfeit 4 (SURF4). The exosomal expression of KIT, and several other proteins identified by MS including hypoxia-inducible factor 1-alpha (HIF1-α) and signal transducer and activator of transcription 1 (STAT1) were then validated in patients. Immunoblotting of patient-derived GDEs isolated from plasma samples confirmed that, consistent with the *in vitro* data, KIT, HIF1-α and STAT1 were localised in patient exosomes. These studies suggest that monitoring for the expression of selected proteins in circulating exosomes could serve as liquid biopsies for rapid evaluation of tumour response and relapse in GIST patients undergoing treatment with IM.

Taken together, these studies demonstrate that proteomics has utility in GIST including identification of new prognostic risk classifiers, mechanisms of IM response and resistance; and circulating biomarkers for evaluation of therapy response and relapse.

### 3.4 Liposarcoma

Liposarcoma (LPS) is a common adult STS subtype, accounting for approximately 20% of all diagnoses (75). They originate from malignantly transformed adipocyte progenitor cells, and are further subdivided into distinct histological and biological subtypes (76). The most common LPS subtypes comprise well-differentiated (WDLPS) and dedifferentiated liposarcoma (DDLPS), while the rarer subtypes are myxoid liposarcoma (MLPS) and pleomorphic liposarcoma (PLPS). WDLPS and DDLPS tend to arise in the retroperitoneum, with delayed symptoms leading to large size at presentation. Histologically, WDLPS closely resemble mature adipose tissue with fibrous septa and variable nuclear atypia, whilst DDLPS is more akin to an undifferentiated or spindle cell sarcoma (77). Although DDLPS can occur *de novo*, it is typically identified as a non-adipocytic sarcoma in proximity to WDLPS and represents progression from pure WDLPS to the higher-grade malignancy. Clinically, WDLPS presents as a locally aggressive neoplasm without metastatic potential, whilst DDLPS has a more aggressive phenotype with a greater propensity for local recurrence and metastases. Despite the variability in clinical behaviour, both WDLPS and DDLPS share similar genetic aberrations displaying a 12q21-15 amplicon creating a ring twelfth chromosome which includes the MDM2 proto-oncogene (*MDM2)* and cyclin dependant kinase 4 (*CDK4)* cell cycle oncogenes (78). The different clinical phenotypes has been explained through analysis of paired WD and DDLPS tissue in which the DDLPS samples contained higher numbers of the aforementioned chromosome 12 amplifications, as well as higher numbers of somatic copy number variations and fusion transcripts (79).

MLPS tend to arise in the deep soft tissue of the extremities with a peak incidence in the fourth and fifth decades of life. Histologically, MLPS demonstrates a mix of uniform oval shaped cells and ring cell lipoblasts on a background of myxoid stroma, with a higher proportion of ring cells correlated with a worse prognosis (80). Unlike WD/DDLPS, MLPS is a translocation driven sarcoma and is characterised by the recurrent translocations t(12,16)(q13;p11), and less commonly t(12,22)(q13;q12), which fuse FUS RNA binding protein (*FUS)* or EWS binding protein-1 (*EWSR1)*, respectively, to DNA damage inducible transcript-3 *(DDIT3)* on chromosome 12. These novel transcription factors inhibit adipocytic differentiation (81).

PLPS represents the least common LPS subtype, but the most clinically aggressive with local recurrence or distance metastases occurring in around 40% of cases (82). Typically occurring in the limbs in those over 50 years of age, PLPS is histologically characterised by variable numbers of pleomorphic lipoblasts on a background of a high-grade pleomorphic sarcoma (83). Given the relative scarcity of PLPS, molecular studies of this subtype are sparse; however, reports suggest a complex karyotype including unpredictable gains and deletions, although deletion of 13q14.2-q14.3, a region including the known tumour suppressor *RB1*, has been observed in 60% of PLPS (84).

Early detection in cancer has been shown to result in vastly improved clinical outcomes (85). One approach to improve rates of early disease detection is in the monitoring of circulating tumour biomarkers detectable through routine blood sampling. Platelets have previously been shown as a potential reservoir for tumour biomarkers (86). In particular, platelets have been noted to specifically sequester angiogenic factors, many of which are involved in pathways known to be upregulated in LPS. In a study by Cervi et al., surface-enhanced laser desorption/ionisation MS, a variant of MALDI MS, was performed for platelet and plasma proteomic profiling in MLPS xenograft mouse models (87). Of note, platelet factor 4 (PF4), a pro-angiogenic protein, was found in the platelets of mice bearing LPS tumours at 120 days post-implantation and not in non-tumour-bearing mice. Further to sustained and elevated PF4 levels observed at day 120, PF4 was also significantly increased at day 19, where mice bore only microscopic, non-palpable, and non-angiogenic tumours. This study highlights PF4 as a promising candidate early disease biomarker, potentially detectable in patients harbouring tumours which would likely go undetected with current imaging modalities.

LPS biomarker proteomic studies have also been undertaken to identify predictors of dedifferentiation in well-differentiated lipogenic tumours. Atypical lipotamous tumour (ALT) is one example of a well differentiated benign tumour which can dedifferentiate and develop into an aggressive malignancy. Currently, anatomical site is the only known predictor for dedifferentiation and there is no reliable molecular predictor available. Identification of such biomarkers may enable patient stratification for those at risk for dedifferentiation, and consequently highlight a subgroup of patients most likely to benefit from more frequent monitoring or potential trials of adjuvant therapy. With this in mind, McClain et al. sought to identify biomarkers predictive of dedifferentiation by comparison of ALT and DDLPS tumours through IHC and 2D-DIGE (88). No significant differentially expressed proteins were identified between the two tumour types, however 6 proteins were found to be differentially expressed in the WDLPS component regions of DDLPS compared to ALT, including selenium binding protein 1 (SELENBP1). Using IHC in an independent cohort of 30 ALT and 28 DDLPS cases, SELENBP1 was confirmed to be expressed at significantly lower levels in WDLPS areas compared to ALT. The role of SELEBP1 under physiological conditions is not fully understood, however it has previously been shown to have a reduced expression in carcinomas, and by extension has been hypothesised to mediate tumour suppressive effects in cancer (89,90). Taken together, the study by McClain et al., reinforces the potential role of SELENBP1 as a candidate tumour suppressor for driving dedifferentiation of mesenchymal tumours such as LPS.

These proof-of-principle studies demonstrate the promise of proteomics in furthering our knowledge about LPS, in particular potential molecular drivers of early tumour angiogenesis and dedifferentiation. However much work remains to be done in order to delineate the biological roles of these proteins and translate these findings into robust clinical applications.

### 3.5 Leiomyosarcoma

Leiomyosarcoma (LMS) account for approximately 10-20% of adult STS diagnoses, and display features consistent with smooth muscle differentiation, histologically showing blunt-ended spindle cells with eosinophilic cytoplasm. LMS are typically observed in middle-aged and older adults and usually occur in the smooth muscle wall of a blood vessel or tubular digestive organ, whilst uterine LMS (uLMS) are often considered a separate entity and occur in the myometrium. Both LMS and uLMS have complex karyotypes with no consistent genetic aberrations at the chromosomal level identified. Despite this complex genomic landscape, published literature has identified more frequent losses involving the tumour suppressor genes *RB1* at the 10q position and phosphatase and tensin homolog (*PTEN)* at 13q (91). Furthermore, whole-exome sequencing analysis of cohorts of LMS and uLMS have confirmed a heterogeneous genomic landscape, but with frequent copy number variation and common alterations in the tumour suppressor genes *RB1, TP53, PTEN* and cadherin-1 (*CDH1)* (92). The Cancer Genome Atlas consortium undertook whole genome sequencing, DNA methylation, messenger RNA and microRNA analysis of 206 STS specimens, including 80 LMS cases, of which 53 were soft tissue LMS and 27 uLMS (93). They reported soft tissue LMS and uLMS to be more similar to each other compared to other STS subtypes, however they identified distinct methylation and mRNA expression profiles between the two LMS groups, with non-uterine LMS displaying a more prominent HIF1α signalling signature compared to uLMS which showed a higher DNA damage response signature.

Transcriptomic studies have revealed three molecular subtypes of LMS; 1 with an mRNA signature mapping mainly to uLMS, and 2 other subtypes of mixed anatomical origins ([93], [94], [95]). Although integrative proteogenomic studies in other cancer types often note a lack of mRNA-protein correlation at the individual protein level, independent proteomic profiling analysis of LMS tumours has also alluded to the presence of three LMS subtypes (96,97). A study by Kirik et al. of 38 LMS cases and 16 undifferentiated pleomorphic sarcoma (UPS) cases analysed by 2D-DIGE, revealed three LMS proteomic subgroups, as well as a fourth UPS-enriched subgroup (96). To provide further in-depth comprehensive analysis of the tumour proteome, 5 samples from each of the identified subgroups were subjected to quantitative analysis by TMT-based ESI-MS/MS. Consequently, 778 proteins were quantified (in at least 1 sample), and the levels of three proteins, VINC, COL6A3, and MYH11 were identified to be capable of discriminating between the 4 groups. These findings demonstrate the utility of proteomics in not only improving the molecular understanding of disease pathophysiology but also in dissecting molecular heterogeneity within and between STS subtypes.

In addition to the profiling of tumour samples, proteomic analysis has also been performed on the uLMS sarcoma cell line MES-SA, with the aim of identifying the molecular factors contributing to the development of resistance to first-line doxorubicin-based chemotherapy. Using 2D-DIGE and MALDI MS, quantitative proteomic profiling was conducted on MES-SA cells treated with doxorubicin (98). This analysis revealed a subset of 87 proteins with altered expression upon short-term acute treatment with doxorubicin. Further to this, to explore acquisition of long-term resistance to doxorubicin, May et al. performed 2D-DIGE and MALDI MS on the doxorubicin resistant MES-SA/DxR cell line, generated by long-term dose escalation treatement of MES-SA with doxorubicin (99). In this study, 208 proteins were found to be differentially expressed between the two cell lines. Gene ontology assessment highlighted pathways associated with metabolism as upregulated in MES-SA/DxR cells. Conversely, proteins related to cell proliferation, gene regulation, and signal transduction were downregulated. Taken together, proteomic profiling of both acute doxorubicin treatment and acquired doxorubicin resistance illustrate a proteomic landscape for how drug resistance develops; from early exposure to doxorubicin to the establishment of a stable doxorubicin resistant cell population. These studies provide a useful resource for the mining of therapeutic targets to tackle doxorubicin resistance in STS, which remains a fundamental unmet clinical need (100,101).

The phenomenon of epithelial-mesenchymal transition (EMT), and conversely mesenchymal-epithelial transition (MET), describe global cellular changes pertaining to cell lineage, and have both been studied extensively across malignancies of epithelial origin ([102], [103], [104], [105]). These phenotypic alterations may result in the development of a more aggressive tumour and thus contribute to an unfavourable prognosis (106). There is accumulating evidence for the presence of EMT/MET within sarcomas, however the biological basis and clinical relevance, particularly that of EMT remain controversial and largely unclear (107). Yang et al. performed a comprehensive transcriptomic and proteomic assessment of 31 LMS and 38 GIST surgical resection samples and revealed a potential role for MET in LMS (108). Using RPPA and transcriptomic whole genome microarray analysis, overexpression of the epithelial marker, E-cadherin, and a concordant reduction in expression of the E-cadherin repressor, Slug, was observed in a subset of LMS patients. Moreover, high E-cadherin expression and low Slug expression was correlated with an improved OS indicating that these proteins may serve as prognostic markers for LMS. As validation of the proteomic data, IHC analysis was performed on the initial cohort, and confirmed that E-cadherin expression correlated with improved OS. Bioinformatic assessment of transcriptomic data also validated this observation, identifying an epithelial gene expression signature in LMS. To assess the functional role MET in LMS, genetic knockdown by RNAi of Slug in the LMS cell line, SK-LMS-1, showed a significant increase in E-Cadherin expression, and a reduction in mesenchymal marker expression (vimetin and N-cadherin). A concomitant reduction in tumour cell proliferation and migration was also observed. Subsequent genetic rescue of Slug by re-expression of the protein rescued these phenotypes, and thus provided mechanistic evidence for Slug-mediated MET in LMS. Increased expression of E-cadherin, and its association with patient survival was not observed in the GIST specimens analysed, reinforcing the distinct biology inherent within different STS histological subtypes.

### 3.6 Malignant rhabdoid tumours

Malignant rhabdoid tumours (MRT), known as atypical teratoid/rhabdoid tumours (AT/RT) when occurring in the central nervous system, are rare and highly aggressive tumours occurring mainly in soft tissues, the kidneys or the brain and typically affecting the paediatric population. They are prone to early metastases, and irrespective of localisation of the primary tumour have a dismal 5-year OS rate of 27-33% (109,110). Histologically, MRTs are characterised by cells with typical rhabdoid morphology including eccentric nuclei, prominent nucleoli, and eosinophilic cytoplasm and inclusions. Cytogenetic and molecular analyses identified a deletion on the long arm of chromosome 22 (22q11.2), with subsequent studies demonstrating a loss-of-function exonic mutation in the SWI/SNF related, matrix associated, actin dependant regulator of chromatin, subfamily b, member 1 *(SMARCB1)* tumour suppressor gene (111). Treatment of MRTs and AT/RTs is multi-modal, with combination chemotherapy, radiotherapy and surgery all having a role in the management of these patients. However, even with intensive therapeutic regimens, the survival outcomes are poor, and is noted to be significantly worse in patients diagnosed before the age of 3 years (112).

Mutational inactivation of *SMARCB1*, a core subunit of the SWI/SNF ATP dependent chromatin-remodelling complex, has been identified as the molecular mechanism driving MRT and AT/RT development (113). Previously, constitutive activation of the kinase AKT has been reported in *Smarcb1* deficient murine cells and hypothesised to contribute to tumorigenic transformation (114). However, the mechanistic basis driving AKT activation upon *Smarcb1* loss is unclear. Accordingly, quantitative phosphoproteomic analysis by MS has been performed to compare a Smarcb1 deficient murine MRT tumour cell line 365, and a Smarcb1 proficient cell line 365 transduced with a *Smarcb1*-containing retroviral vector (115). This analysis identified 3655 proteins, of which 407 showed differential phosphorylation status across 616 phosphorylation sites upon ectopic expression of Smarcb1. In order to assess specific kinase involvement driving the observed phosphorylation alterations, kinase target enrichment of the dataset was performed. Consistent with previous findings, targets of AKT, including Bad, Cdc25B, and TSC2, were significantly enriched in Smarcb1-deficient cells. A lesser enrichment of ERK1/2 and JNK1 targets was also observed. Further analysis highlighted EGFR phosphorylation at Y1197, an autophosphorylation site associated with receptor activation, and phosphorylation of several of its downstream effectors, ERK2 (T183), JUN (S63), and MYC (T58) upon loss of Smarcb1. Subsequently, the signalling effects of EGFR activation in murine rhabdoid cell lines 167 and 365 was assessed by treatment with gefitinib, an EGFR-specific inhibitor and lapatinib, a dual target EGFR/ErB2 inhibitor. AKT phosphorylation was reduced upon treatment with either drug, implicating the EGFR signalling axis in the AKT activation observed in Smarcb1 deficient cells, highlighting the potential use of EGFR inhibitors in the treatment of MRT and AT/RT.

Several tyrosine kinase inhibitors (TKIs) are approved or under investigation for the treatment and management of sarcoma. Two such TKIs are; Pazopanib (Paz), an inhibitor of c-KIT, PDGFR, fibroblast growth factor receptor-1 (FGFR1), and vascular endothelial growth factor receptor (VEGFR), and Dasatinib (Das), an inhibitor of PDGFR, BCR-ABL, and Src family kinases. Wong et al., undertook a tyrosine phosphoproteomic MS analysis to assess the signalling profiles of the MRT cell line A204, and their Paz-resistant (PazR), and Das-resistant (DasR) counterparts which were generated by long-term administration with escalating doses of each respective TKI (116). This analysis revealed that the TKI-sensitive parental A204 cells displayed higher phosphorylation of PDGFRα and FGFR1 compared to DasR and PazR cells. Moreover, cells resistant to PDGFRα inhibition were shown to be sensitive to FGFR1 inhibition. Dual inhibition of both PDGFRα and FGFR1, using either a combination of FGFR1 inhibitor and Paz or Das, or the dual inhibitor Ponatinib, increased cellular apoptosis, Thus inhibition of PDGFRα and FGFR1 was demonstrated as a viable method to overcome TKI resistance. Vyse et al. built on this study to compare the signalling pathways between A204 cells that has acquired resistance to either Paz or Das (117). By employing global phosphoproteomic analysis in the A204 parental, PazR and DasR cells the authors showed that a surprisingly low fraction of the phosphorylation sites quantified were altered upon acquisition of Paz and Das resistance (6% and 9.7% respectively). In PazR cells, upregulation of cytoskeletal pathway phosphorylation was observed. Specifically, there was significant upregulation of actin binding proteins, LIM domain containing proteins and calponin homology domain (CDH) containing proteins. In contrast, upregulated phosphorylation of the insulin receptor/insulin growth factor 1R (IGF-1R) pathway was seen in DasR cells. This included proteins acetyl-CoA carboxylase alpha (ACACA), A-Raf proto-oncogene (ARAF), fatty acid synthase (FASN), insulin receptor substrate 1 (IRS1), protein kinase CAMP dependent protein 1B/2B (PRKAR1B/2B), ribosomal protein S6 kinase A1/B1 (RPS6KA1/B1) and SHC-transforming protein (SHC), all of which comprised an integrated protein-protein interaction network that forms a large module of the insulin receptor-signalling pathway. Supporting previous observations that Paz and Das resistance develop via different mechanisms (116), minimal overlap is observed between the phosphoproteomic datasets. When integrated, only a small fraction phosphosites were upregulated (2.8%) or downregulated (1.9%) in both resistant cell lines. This study provided a comprehensive view of signalling alterations acquired as a result of drug resistance, and acts as a future guide for candidate targets for salvage therapies to overcome drug resistance and achieve durable responses in patients.

### 3.7 Myxofibrosarcoma

Myxofibrosarcoma (MFS) is one of the most common STS subtypes that affect the elderly population, with an equal sex distribution, and an average age at diagnosis of 66 years (118). MFS typically affect the extremities and limb girdles, with an infiltrative growth pattern which leads to deep-seated tumours and a high risk of post-surgical local recurrence due to borderless extension of atypical cells along vascular and fascial planes (119). Although cytogenic analyses of MFS are scarce, it has been shown that they harbour a high level of genomic variability, ranging from normal karyotypes to highly complex karyotypes with copy number variations, clonal and nonclonal aberrations (118). More recent studies have demonstrated a non-random loss of chromosome 9, with deletion of the methylthioadenosine phosphorylase *(MTAP)* and *CDKN2A/CDKN2B* genes (9q21.3) associated with increased aggressiveness in myxofibrosarcoma (120,121). Although surgical excision remains the mainstay of treatment, the infiltrative pattern of MFS allows considerable spread beyond the gross tumour margins necessitating as large a resection as possible. Even with this treatment paradigm, local recurrences are common occurring in more than 50% of MFS cases with the recurrent lesion frequently a higher-grade than the primary tumour (122,123).

To probe for drivers of invasiveness in MFS and identify potential biological mechanisms amenable to therapeutic inhibition, 2D-DIGE has been employed to profile MFS tumours of differing intrinsic invasiveness (124). Invasiveness status was determined by inspection of tumour margins by magnetic resonance imaging (MRI) assessment for a MFS-characteristic infiltrative, tail-like pattern (122). Comparative assessment of 6 invasive tumour samples versus 5 non-invasive specimens revealed 47 proteins as differentially expressed between the two groups. Of note, Discoidin, CUB And LCCL Domain-Containing Protein 2 (DCBLD2) was significantly upregulated within the invasive patient subgroup. DCBLD2 has previously been associated with tumourigenic activity in several other cancer types, and accordingly the observed positive correlation between high DCBLD2 expression and increased MFS invasiveness was selected for further assessment (125,126). IHC of 21 additional MFS cases confirmed the association of DCBLD2 expression with tumour invasiveness. Moreover, DCBLD2 as a predictor for an invasive phenotype was shown by the authors to have high specificity of 87.5%, highlighting its promise as a robust means of stratifying invasion versus non-invasive lesions.

### 3.8 Alveolar soft part sarcoma

ASPS is an ultra-rare sarcoma subtype, accounting for around 1% of all STS diagnoses, with a female predominance and typically occurring in the third decade of life (127). Although indolent in its early stages, ASPS has a preponderance for early metastasis, with 55% of cases only diagnosed at stage IV disease with a 5-year OS of 61% compared to 87% in those patients with localised disease (128). ASPS is characterised by the pathognomonic unbalanced, non-reciprocal translocation t(X;17)(p11:q25), fusing the alveolar soft part sarcoma chromosome region candidate 1 (*ASPSCR1)* gene on chromosome 17 to the transcription factor binding to IGHM enhancer 3 (*TFE3)* gene on the X chromosome (129). In the *ASPSCR1-TFE3* fusion there is preservation of the DNA binding and transcriptional activation domains of *TFE3*, which is hypothesised to be the oncogenic driver of ASPS through ubiquitous and high-level expression of its fusion partner *ASPSCR1* (130).

Kubota et al. conducted a 2D-DIGE MS study on 12 archival ASPS cases which revealed a subset of 145 proteins that were differentially expressed between matched normal and tumour samples (131). Of these proteins, Protein SET, an inhibitor of the tumour suppressor protein phosphatase 2 (PP2A), was explored further. Overexpression of SET in ASPS was confirmed by IHC in an expanded cohort of 15 ASPS cases, relative to normal tissue. Moreover the involvement of SET in tumourigenesis was investigated by genetic knockdown in the ASPS cell line ASPS-KY. Silencing of SET resulted in reduced cell proliferation, invasion and migration, implicating this protein as a driver of tumourigenesis in ASPS. Furthermore, this observation was phenocopied upon addition of FYN720, a PP2A activator used in the treatment of multiple sclerosis, providing mechanistic evidence for PP2A and SET involvement in ASPS, and highlighting the potential clinical utility of FYN720 for the treatment of ASPS patients.

### 3.9 Integrative proteomics of multiple sarcoma subtypes

Beyond proteomic profiling of individual sarcoma subtypes, several reported studies have undertaken integrative analysis of multiple histological subtypes. Assessment of different subtypes enables generation of statistically significant cohort sizes, both for discovery and validation studies. Moreover, integrative analysis allows for similarities and differences between subtypes to be assessed; offering a greater understanding of the biology of these cancers than is possible when studying a single subtype alone. The largest integrative proteomic study to date has been performed by The Cancer Genome Atlas Research Network (93). Using RPPA, the expression levels of 192 proteins was assessed across a cohort of 173 specimens, comprised of 60 LMS, 46 DDLPS, 41 UPS, 15 MFS, 6 SS, and 5 MPNST cases. Analysis of this dataset led to the identification of 5 stable clusters of molecular groups, 1 enriched for LMS (48 out of 53 samples were LMS), and the remaining 4 occupied by a mixture of subtypes. Notably, the LMS enriched cluster signature exhibited lower apoptosis pathway activity, higher PI3K/AKT pathway activity, and higher expression of oestrogen and progesterone receptors compared to the other 4 clusters. Further inspection of the data revealed that AKT activity was enriched in non-uterine LMS cases, and higher hormone receptor expression attributable to uLMS cases.

In another large comparative study of multiple STS subtypes, Lou et al., utilised MALDI MS to characteris MFS, osteosarcoma, LMS and UPS. The authors profiled 52 high grade osteosarcoma, LMS and UPS cases, and found that proteomic profiles provided clear discrimination between subtypes (132). The authors also demonstrated that the expression of 9 proteins, including the proteasome complex subunit 1 (PSME), was associated with poorer survival in all subtypes except osteosarcoma. More recently, these data were complimented by metabolic assessment of 10 MFS, 7 osteosarcoma, 8 LMS, and 8 UPS cases (133). As with protein profiling, MALDI MS was employed for metabolic analysis, and the resultant metabolite profiles revealed specific signatures mapping to each subtype. When combined with clinical data, these data highlighted inositol cyclic phosphate and carnitine as metabolites correlating with poorer OS and metastasis free survival (MFS). Both carnitine and inositol cyclic phosphate have been previously implicated in tumourigenesis; the former reported in colon cancer, and inositol triphosphate (IP3) signalling, to which inositol cyclic phosphate contributes, reported as aberrantly active in acute myeloid leukaemia (134,135).

Such studies demonstrate the utility of integrative proteomics to investigate multiple subtypes and show distinct molecular subgroups of distinct biology within and between subtypes. Furthermore, these studies have identified the expression of several proteins and metabolites which are indicative of disease progression. These studies demonstrate proof-of-principle and highlight the promise of large-scale integrative analysis of multiple sarcoma subtypes to bridge the gap in our knowledge of disease etiology and pathophysiology.

## 4 Challenges and future perspectives

In recent years, the use of proteomics to study sarcomas has grown to encompass several different methodologies spanning an increasing number of histological subtypes, including several ultra-rare ones. Despite these advances, proteomic research in sarcomas remains in its infancy. At present proteomics has been utilised primarily for biomarker discovery and global profiling of sarcomas. The full potential of proteomics however extends far beyond these applications, and holds huge potential for accelerating sarcoma research.

The sarcoma community faces several key challenges in clinical disease management. Curative treatments in sarcomas are rare, with 50% of patients eventually relapsing with incurable metastatic disease following surgical resection of localised tumours (136,137). Improvements have previously been hindered by a ‘one size fits all’ approach to therapy, whereby standard chemotherapy approaches are used irrespective of sarcoma subtype. Due to the highly heterogeneous nature of sarcomas, response rates to these chemotherapies varies greatly both between and within sarcoma subtypes. Accordingly, there is an urgent need for the development of more effective targeted therapeutic regimens and biomarkers for robust patient stratification based on likelihood of treatment effectiveness. In addition, there is an urgent need for additional prognostic biomarkers that predict for risk of disease relapse following surgery in the setting of localised disease. Another key clinical issue is the emergence of drug resistance to both chemotherapy and targeted agents. Depending on the histological subtype, patients can frequently experience an initial period of successful disease management. Unfortunately, treatments responses are often shortlived and drug resistance develops. At this stage, options for disease management are limited and disease progression often ensues.

These challenges underscore the gap in knowledge in our understanding of the biology underpinning sarcoma pathogenesis and therapy response. Specifically, there are three areas of unmet need where proteomics could play a central role in addressing. Firstly, we do not fully understand the molecular mechanisms driving sarcoma development and progression. Secondly, there are no validated biomarkers or molecular signatures capable of predicting disease relapse and treatment response, and thirdly, understanding of the mechanisms of drug resistance is lacking. For instance, global profiling by proteomics facilitates the deep annotation and characterisation of biological pathways associated with sarcoma disease biology and drug resistance which can inform subsequent functional studies in preclinical models of disease. In addition, phosphoproteomics has particular utility in revealing the key phosphorylation-mediated signalling nodes within biological networks in sarcomas driven by aberrant kinase signalling (14). Beyond the use of proteomics alone, the benefits of Omics data integration are being realised. Large-scale multi-omics studies, such as those performed by The Cancer Genome Atlas (TCGA) and The Clinical Proteome Tumour Analysis Consortium (CPTAC), arguably provide the most comprehensive picture of tumour status, and have huge potential if expanded to large cohorts of sarcoma patients. In the era of big data, as such datasets expand, there will be an increasing need for the use of next generation machine learning and artificial intelligence (AI) approaches for data mining to identify multi-feature biomarkers that may be predictive of disease susceptibility, recurrence, and prognosis. This strategy should extend beyond just the analysis of profiling data alone, and incorporate other clinical measures such as imaging and digital pathology ([138], [139], [140], [141], [142], [143], [144]). We anticipate that such holistic data integration approaches will undoubtedly drive new discoveries in the sarcoma field moving forward.

An exciting future application of proteomics is in correlative sciences within the context of sarcoma clinical trials, to drive the realisation of personalised molecular medicine in these diseases. Characterising and mapping of the proteomic changes in tissue and blood during disease progression, on treatment, upon the acquisition of drug resistance, and at disease recurrence will provide unparalleled insights into tumour behaviour over the clinical course of disease. Our view is that the routine integration of proteomics into correlative sciences within clinical trials will ultimately bolster our ability for the comprehensive evaluation of disease biology in real time with future impact in early detection and disease monitoring.

## 5 Conclusion

In summary, we have presented a comprehensive review of the current status of the use of proteomics for the analysis of a range of different sarcoma subtypes and outline potential future directions for this exciting area of research. We fully expect that new initiatives that integrate proteomics into multi-omic studies and clinical trials will pave the way for rapid translation of laboratory discoveries into the clinic and ultimately have an impact in improving clinical management and outcomes of sarcoma patients.

## Transparency document

Transparency Document
