# Supplementary material for: Proteomic research in sarcomas – current status and future opportunities
Source: Semin Cancer Biol. 2020 Apr;61:56–70. doi: 10.1016/j.semcancer.2019.11.003 (PMC7083238; doi:10.1016/j.semcancer.2019.11.003)
Supplement: Transparency Document [file mmc1.zip › COI_YSCBI RLJ.pdf]

ELSEVIER

Seminars in Cancer Biology

Conflict of Interest Policy

Article Title: **Proteomic research in sarcomas current status and future opportunities**

Author name:
Robin L Jones

Declarations

Seminars in Cancer Biology requires that all authors sign a declaration of conflicting interests. If you have nothing to declare in any of these categories then this should be stated.

Conflict of Interest
A conflicting interest exists when professional judgment concerning a primary interest (such as patient's welfare or the validity of research) may be influenced by a secondary interest (such as financial gain or personal rivalry). It may arise for the authors when they have financial interest that may influence their interpretation of their results or those of others. Examples of potential conflicts of interest include employment, consultancies, stock ownership, honoraria, paid expert testimony, patent applications/registrations, and grants or other funding.

Please state any competing interests

Consultant for: Adaptimmune, Blueprint, Clinigen, Eisai, Epizyme, Daichii, Deciphera, Immunedesign, Lilly, Merck, Pharmamar, Tracon, UptoDate.

Funding Source
All sources of funding should also be acknowledged and you should declare any involvement of study sponsors in the study design; collection, analysis and interpretation of data; the writing of the manuscript; the decision to submit the manuscript for publication. If the study sponsors had no such involvement, this should be stated.

Please state any sources of funding for your research

Signature (a scanned signature is acceptable, but each author must sign)

Print name

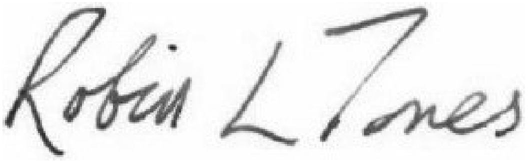

Robin L Jones
