# Supplementary material for: Proteomic research in sarcomas – current status and future opportunities
Source: Semin Cancer Biol. 2020 Apr;61:56–70. doi: 10.1016/j.semcancer.2019.11.003 (PMC7083238; doi:10.1016/j.semcancer.2019.11.003)
Supplement: Transparency Document [file mmc1.zip › COI_YSCBI_CW.pdf]

ELSEVIER

Seminars in Cancer Biology

Conflict of Interest Policy

Article Title:<br>Proteomic research in sarcomas current status and future opportunities	Author name:<br>Christopher Wilding

Declarations

Seminars in Cancer Biology requires that all authors sign a declaration of conflicting interests. If you have nothing to declare in any of these categories then this should be stated.

Conflict of Interest

A conflicting interest exists when professional judgment concerning a primary interest (such as patient's welfare or the validity of research) may be influenced by a secondary interest (such as financial gain or personal rivalry). It may arise for the authors when they have financial interest that may influence their interpretation of their results or those of others. Examples of potential conflicts of interest include employment, consultancies, stock ownership, honoraria, paid expert testimony, patent applications/registrations, and grants or other funding.

Please state any competing interests

None.

Funding Source

All sources of funding should also be acknowledged and you should declare any involvement of study sponsors in the study design; collection, analysis and interpretation of data; the writing of the manuscript; the decision to submit the manuscript for publication. If the study sponsors had no such involvement, this should be stated.

Please state any sources of funding for your research

Funding received from Sarcoma UK, Wellcome Trust, Royal Marsden Cancer Charity, Cancer Research UK and the Cynthia Marsh Memorial Fund.

The sponsors had no involvement in generation of this manuscript.

Signature (a scanned signature is acceptable, but each author must sign)

____

Print name

___Christopher Wilding_____
